# Seed dispersal by macaws shapes the landscape of an Amazonian ecosystem

**DOI:** 10.1038/s41598-017-07697-5

**Published:** 2017-08-07

**Authors:** Adrián Baños-Villalba, Guillermo Blanco, José A. Díaz-Luque, Francisco V. Dénes, Fernando Hiraldo, José L. Tella

**Affiliations:** 1 Department of Molecular Biology and Biochemical Engineering, University Pablo de Olavide Ctra, Utrera km 1, E- 41013 Sevilla, Spain; 2 Department of Evolutionary Ecology, Museo Nacional de Ciencias Naturales, CSIC. José Gutiérrez Abascal 2, 28006 Madrid, Spain; 3 Bolivian Parrots Research and Conservation Foundation (CLB), Avda. Mariscal Sta. Cruz 5030, Santa Cruz de la Sierra, Bolivia; 4 Department of Conservation Biology, Estación Biológica de Doñana, CSIC. Américo Vespucio s/n, E-41092 Sevilla, Spain

## Abstract

Seed dispersal is one of the most studied plant–animal mutualisms. It has been proposed that the dispersal of many large-seeded plants from Neotropical forests was primarily conducted by extinct megafauna, and currently by livestock. Parrots can transport large fruits using their beaks, but have been overlooked as seed dispersers. We demonstrate that three macaws (*Ara ararauna*, *A*. *glaucogularis* and *A*. *severus*) are the main dispersers of the large-seeded motacú palm *Attalea princeps*, which is the biomass-dominant tree in the Bolivian Amazonian savannas. Macaws dispersed fruits at high rates (75–100% of fruits) to distant (up to 1200 m) perching trees, where they consumed the pulp and discarded entire seeds, contributing to forest regeneration and connectivity between distant forests islands. The spatial distribution of immature palms was positively associated to the proximity to macaws’ perching trees and negatively to the proximity to cattle paths. The disperser role of livestock, presumably a substitute for extinct megafauna, had little effect due to soil compaction, trampling and herbivory. Our results underscore the importance of macaws as legitimate, primary dispersers of large-seeded plants at long distances and, specifically, their key role in shaping the landscape structure and functioning of this Amazonian biome.

## Introduction

Plant-visiting animals play significant roles in the ecological and evolutionary dynamics of plant communities and ecosystems. In particular, frugivorous vertebrates are considered key elements in the integrity of ecosystems by promoting large-scale exchange of genetic information through seed flow^1^. By influencing the spatial distribution and demography of the plants they feed on, seed dispersers may shape the vegetal landscapes and contribute to the resilience and natural regeneration of ecosystems^1, 2^. In fact, most terrestrial ecosystems depend on animals for seed dispersal, especially in tropical and subtropical forests where up to 70% of the total of woody species are dispersed by vertebrates^1, 3^. Among them, the largest frugivores have been highlighted as a central component of dispersal networks by their major contribution to the dispersal of large-seeded plant species^4–6^.

Conversely, resources provided by fruiting plants influence the ecology, fitness and population size of their consumers and mutualist dispersers^3^. This feedback between the resources provided by plants and the consumers’ use and payoff in the form of mutualistic services is expected to become functionally adjusted to optimize the outcome of the interaction in terms of enhanced fitness for both partners^7^. The dispersal of plants with fleshy fruits whose seeds are moved when consumed by animals represents a well-known example of mutual benefits leading to the evolution of fruit features and diversity shaped by the size, foraging behavior and other traits of the dispersers^8, 9^. Therefore, the loss or numerical reduction of the dispersers in the ecosystems may have rapid ecological and evolutionary consequences in the plants, including a reduced seed dispersal and reduction of seed size^10^. In other cases, mutualistic interactions may lose their current function before the actual disappearance of the species due to human activities in many extant ecosystems^11^.

A particularly extreme case of dispersal limitation is thought to involve large-sized fruits of several tropical plants presumably dispersed in the past by extinct megafauna^12, 13^. In Neotropical forests many plants have large fruits and other features representing the so-called megafaunal syndrome, including those in the genera *Attalea*, *Pouteria*, *Genipa*, etc^13^. The seed dispersal role presumably conducted in the past by extinct megafauna has been argued as being completely lost or partially conducted by other wild large-bodied potential dispersers, such as tapir *Tapirus terrestris*, or replaced by introduced domestic animals like cows, pigs and horses^12–15^. The role of these alternative dispersers may be limited due to hunting pressure on wild large-bodied dispersers^16–21^ and the fact that the novel domestic dispersers, despite increased abundance in natural environments^22^, have not evolved with the plants they consume and thereby may fail to disperse seeds to suitable recruitment sites (e.g. livestock depositing seeds in paths where soil compacted by frequent trampling may reduce seeding recruitment^23^).

To be functionally effective, dispersal of large seeds from fleshy-fruited plants is often assumed to require seed ingestion and defecation or regurgitation after transit within the disperser digestive tract^3, 24^. This limits dispersal of large seeds to large-sized dispersers, and thereby dispersal has been argued to be constrained by gape or mouth size in many fruit-vertebrate dispersal interactions^25, 26^. This implies that even large avian frugivores like curassows, guans (Cracidae) and toucans (Ramphastidae), or mammals such as tapir (Tapiridae) can only disperse the smallest seeds of many large-seeded plants. Other plants produce seeds that are larger or at the size limit that extant birds or mammals apparently can ingest and defecate^12^. As a consequence, some authors hypothesized that these fruit and seed traits do not represent present-day adaptations, but past ones to currently extinct dispersers, thus appealing to large terrestrial megafauna of the Pleistocene to explain these so-called dispersal anachronisms^12, 13, 27^. However, many large seeds of fleshy and dry fruits can be dispersed via transportation in the mouth, beak or feet by large birds, rodents, carnivore mammals, etc.^28^ without requiring ingestion and defecation, i.e. stomatochory and synzoochory^29^. In particular, parrots have the capacity to transport large fruits and seeds using their beaks or feet, and disperse them over long distances^30–33^. Traditionally, parrots have been considered plant antagonists as seed predators^34–38^, and have not been included in the analysis of mutualistic dispersal interactions^39, 40^. Recent studies have challenged these assumptions by highlighting the importance of parrots as primary ectozoochorous and endozoochorous agents of multiple plant species^31, 41–43^, thus suggesting that this group has been largely overlooked regarding their role in the function and maintenance of tropical ecosystems^30^.

**Figure 1:**
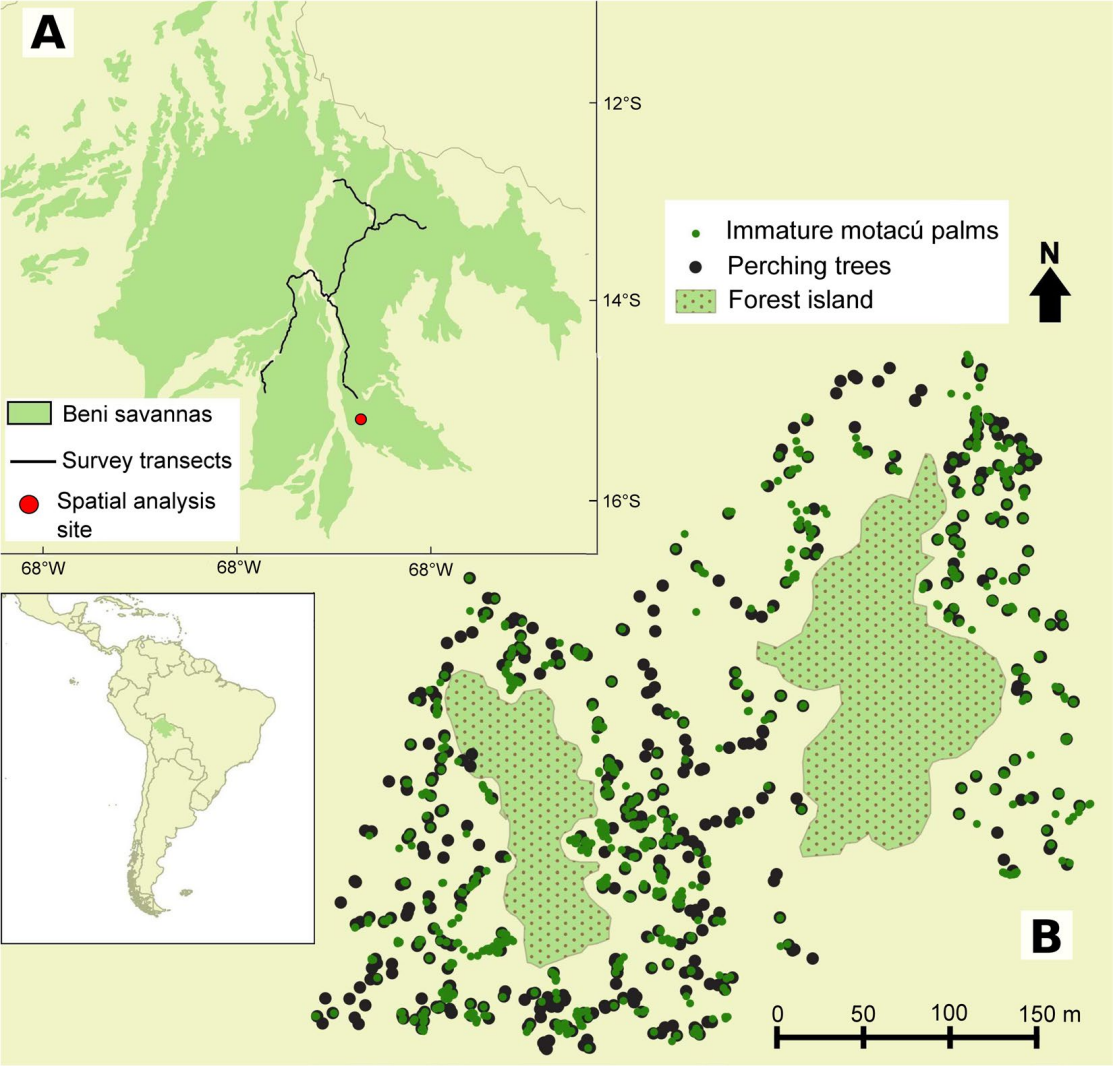
Study area. (**A**) The Amazonian biome of Beni savannas (green) with the survey transects (black lines) and the location of the spatial analysis site (red point). (**B**) The spatial analysis site with the distribution of forest islands (dotted green area), perching trees (black points) and immature motacú palms (green points, point size is proportional to the number of overlapping points). Maps generated with QGIS software v2.12.3, Quantum GIS Development Team (2016). Quantum GIS Geographic Information System. Open Source Geospatial Foundation Project. http://qgis.osgeo.org.

In this study, we examined plant-seed disperser mutualisms in the Amazonian ecosystem of Beni savannas, Bolivia (Fig. 1), by focusing on the relative importance of parrots as dispersers of the large-seeded motacú palm (*Attalea princeps*), a dominant plant species shaping the ecosystem structure in forest islands. Mature forest islands, characteristic of this ecosystem, are dominated by this large-seeded palm^44–46^ and, like in similar Amazonian ecosystems^47, 48^, it represents an important food resource and nesting site for many species, including parrots^45^. The motacú palm has a fleshy fruit and a large seed thought to have been adapted for dispersal by extinct Pleistocene megafauna^12, 13^. However, there is evidence that some parrots consume the pulp and discard the seeds of this species^49^, and that they can move entire fruits in their beaks over large distances^31^.Therefore, we predicted that parrots (especially the larger-bodied macaws) act as legitimate dispersers of this palm species. For such a goal, we evaluated the effect of potential bird and mammal disperser species while accounting for their abundance and considering their fruit consumption and seed dispersal rates. Moreover, we modeled the spatial distribution of immature motacú palms potentially generated as a result of the seeds dispersed by macaws and cattle, which resulted the main dispersers of this species. Our results challenge the seed dispersal anachronism^12, 27^ by showing that macaws are currently the main dispersers of the motacú palm, thus contributing to shaping the landscape structure and function of this Amazonian biome.

## Results

### Seed dispersal

Combining direct observations with camera traps, we recorded a total of 1196 foraging observations of 18 bird and mammal species on 26 plant species, of which fruits of the motacú palm are most commonly dispersed (Table 1).

**Table 1:** Number of individuals, trophic interactions events and plant species involved in the observations of each frugivorous species recorded feeding on large fruits or seeds in the Beni biome.

Species (Family)	N° individuals	N° trophic interactions	N° plant species	N° Seed dispersions (type of dispersal)	Plant species dispersed (n)	Observation method
**Parrots** (**Psittacidae**)						
*Amazona aestiva*	9	22	4	0		DD
*Ara ararauna*	36	99	5	28 (primary)	*A*. *princeps* (28)	DD
*Ara chloropterus*	13	30	3	0		DD
*Ara glaucogularis*	48	86	4	54 (primary)	*A*. *princeps* (54)	DD
*Ara severus*	123	267	12	150 (primary)	*A*. *prínceps* (122), *A*. *totai* (11), *C*. *lilloi* (1), *G*. *ulmifolia* (2), *S*. *sancona* (14)	DD
*Eupsittula aurea*	37	73	9	0		DD
*Psittacara leucophtalhmus*	12	35	2	0		DD
*Aratinga weddellii*	130	135	6	0		DD
*Brotogeris chiriri*	18	40	3	0		DD
*Pionus menstruus*	3	7	1	0		DD
*Primolius auricollis*	3	9	4	2 (primary)	*C*. *lilloi* (1), *S*. *sancona* (1)	DD
**Other frugivores**						
*Cyanocorax cyanomelas* (Corvidae)	2	2	1	2 (secondary)	*A*. *princeps* (2)	DD
*Alouatta caraya* (Atelidae)	4	8	1	0		DN
*Saimiri boliviensis* (Cebidae)	12	16	2	0		CT, DD
*Dasyprocta punctate* (Dasyproctidae)	2	12	1	0		CT
*Euphractus sexcinctus* (Dasypodidae)	1	2	1	2 (secondary)	*A*. *princeps* (2)	CT
*Sciurus ignitus* (Sciuridae)	1	1	1	1 (secondary)	*A*. *princeps* (1)	DD
*Bos taurus* (Bovidae)	143	342	2	328 (secondary)	*A*. *princeps* (137), *A*. *totai* (191)	CT

All seed dispersions are stomatochorous dispersal events (both primary and secondary), except for cow *Bos taurus* that refers to secondary endozoochorous dispersal events. The number of seed dispersals of each plant species conducted by each frugivorous species is also shown. Observation methods differ in sampling effort: Direct-Diurnal (DD) = 420 hours, Direct-Nocturnal (DN) = 60 hours, Camera trap (CT) = 2320 hours.

**Figure 2:**
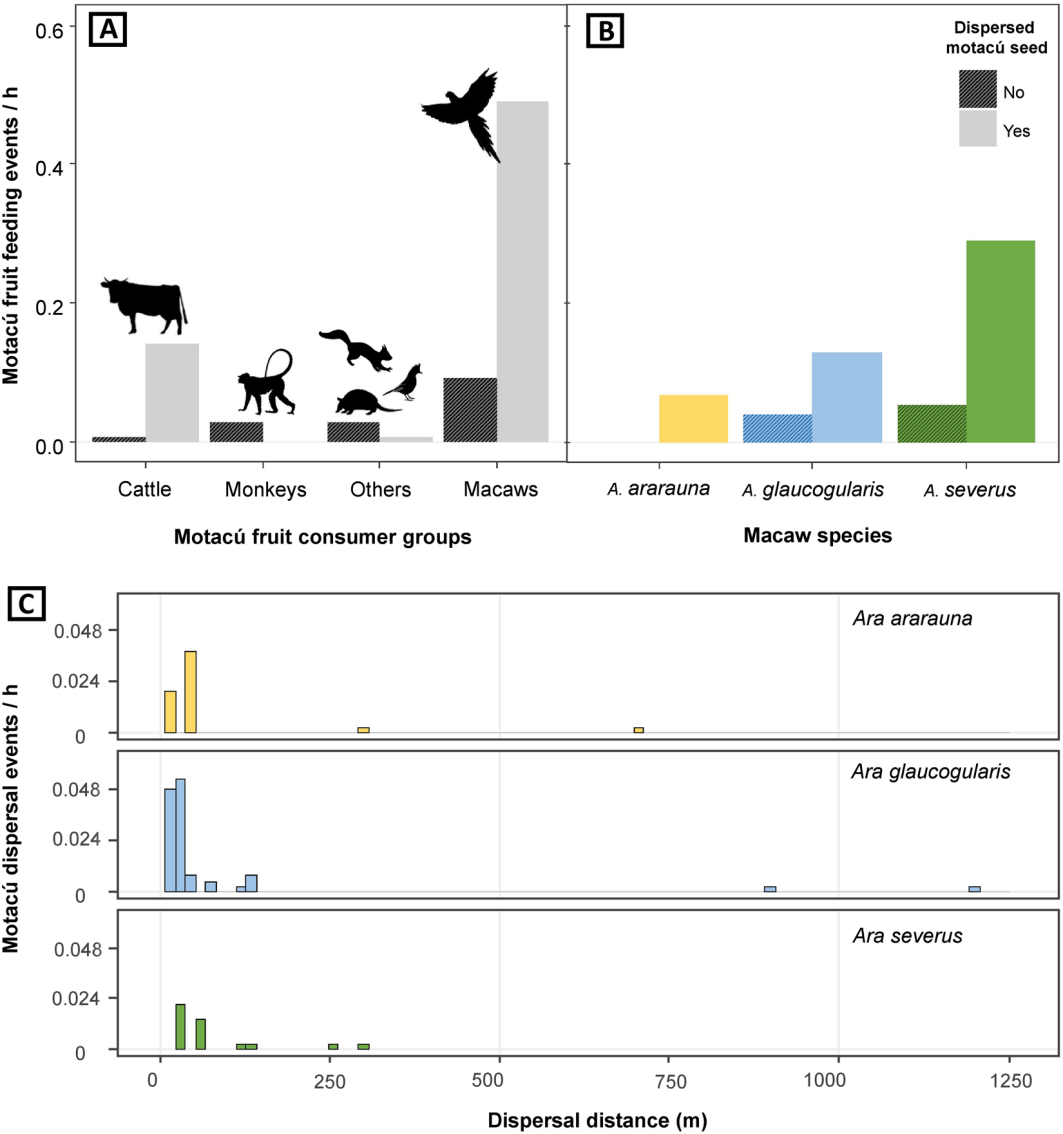
Consumption and seed dispersal rates of motacú palm. (**A**) Differential fruit consumption and seed dispersal rates of each animal group, (**B**) differential fruit consumption and seed dispersal rates of macaw species with some seed dispersion observed, and (**C**) frequencies of seed dispersal distances by each macaw species.

**Figure 3:**
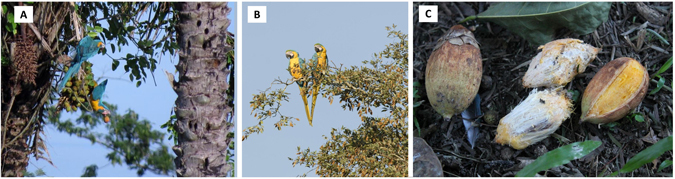
Dispersal of motacú fruits by macaws. (**A**) Two blue-throated macaws *Ara glaugogularis* feeding on a motacú palm; note the individual with a fruit in the beak before flying and dispersing the fruit until a perching place. (**B**) Two blue-and-yellow macaws *Ara ararauna* resting in a perching tree where they typically handle the dispersed fruits. (**C**) One ripe motacú fruit (left), two fully defleshed (center) and one partially defleshed (right) fruit found under perching trees after being dispersed and handled by macaws. Note macaws just eat the pulp, leaving intact the nut which contains viable seeds from ripe fruits. Photographs taken by J.A. Díaz-Luque –Fundación para la Conservación de los Loros de Bolivia (**A**,**C**) and J.L. Tella (**B**).

Centering on the motacú palm, macaws and cattle were the most important dispersers in quantitative terms (Fig. 2a). Cattle dispersed motacú by endozoochory, ingesting the whole fruits that fallen to the ground under mother palms at a rate of ca. 1.8 fruits/h (Fig. 2a). The combined action of three species of macaws (*Ara ararauna*, *Ara glaucogularis*, and *Ara severus*) rendered a dispersal rate almost three times higher (Fig. 2a), although dispersal rates varied among these species (Fig. 2b). Macaws always dispersed by stomatochory, picking the fruit from the palm (Fig. 3a) and carrying it in the beak until a distant perching tree to handle and consume it. From our observations, it seems that these large-bodied macaws have difficulties feeding on the pulp while perching on the unstable motacú pendulous infrutescences (Fig. 3a), which forces them to remove fruits, one at a time, and fly to suitable stable perches (Fig. 3b) to eat them on each feeding occasion. From the 242 fruits picked by macaws, 38 (15.7%) were discarded undefleshed, falling under the mother palm, and 204 (84.3%) were dispersed. Macaws defleshed all the dispersed fruits, consuming partially or completely the pulp and always wasting the intact seed (Fig. 3c). All seeds dispersed were from ripe fruits. The distances to which seeds were dispersed varied among macaw species, with median distances ranging between 29 m for *A*. *glaucogularis* and 51 m for *A*. *ararauna*, with observed long-distance dispersal events reaching up to 1200 m (Fig. 2c). Other much more scarcer potential disperser species were recorded actively dispersing motacú seeds secondarily by stomatochory only five times, twice by jays (Aves: Corvidae) and once by a squirrel (Mammals: Sciuridae) observed during daylight, and twice by an armadillo (Mammals: Dasypodidae) recorded by nocturnal camera trapping (Table 1). In addition, two primate species potentially acting as primary dispersers and a large rodent (*Dasyprocta punctate*, Dasyproctidae) potentially acting as secondary disperser were recorded foraging on motacú fruits but not dispersing their seeds (Table 1). No presence of tapirs was recorded during this study.

### Macaw density and net contribution to motacú seed dispersal

**Figure 4:**
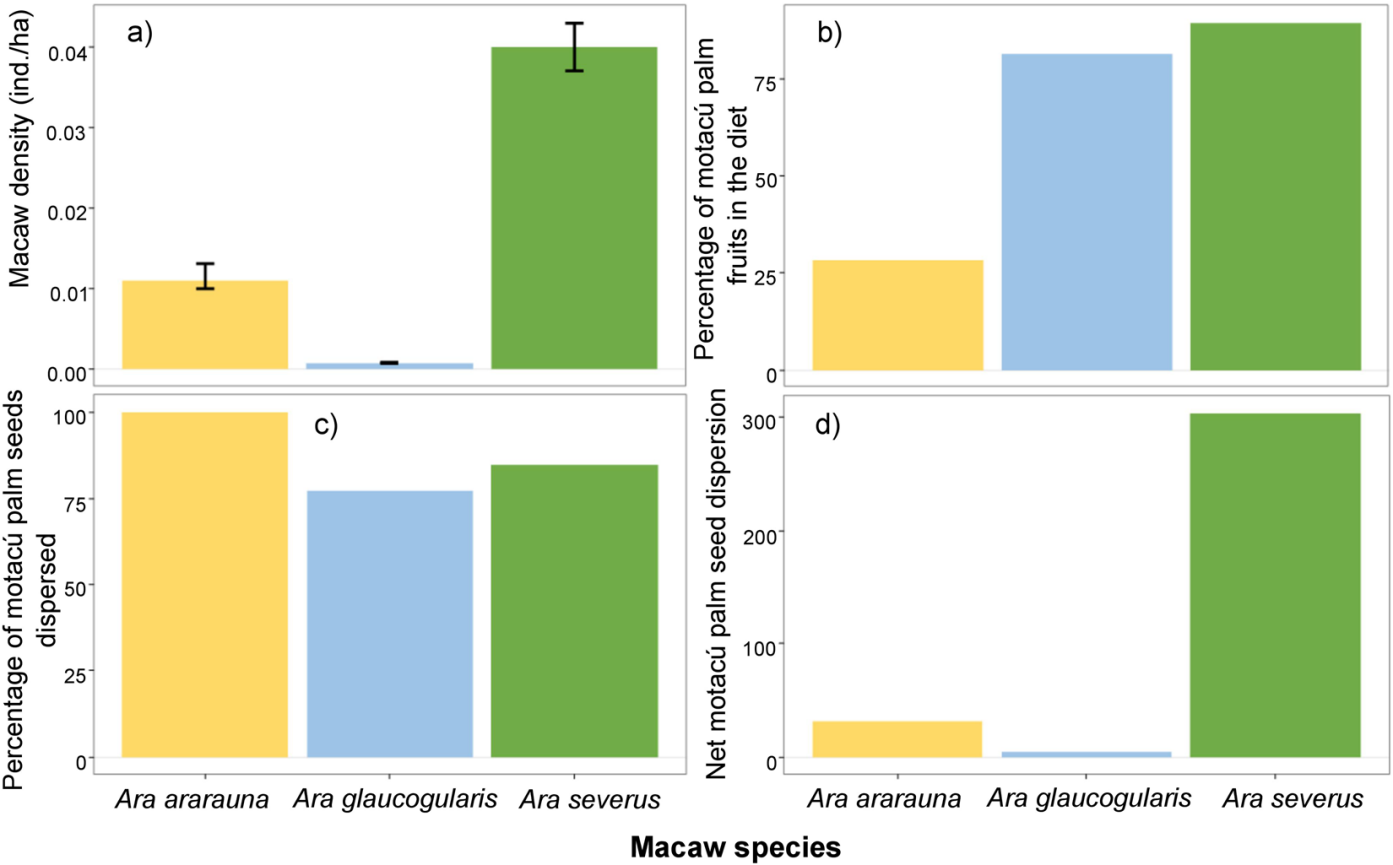
Dispersal contribution of each macaw species. (**a**) Observed macaw density (mean ± 95% CI individuals/ha); (**b**) percentage of motacú palm in diet; (**c**) percentage of dispersed among handled motacú palm seeds; (**d**) net motacú palm seed dispersal by macaw species (Macaw density * % motacú palm fruits in the diet * % motacú palm seeds dispersed).

The three macaw species responsible for most of the dispersals of motacú palm seeds (Fig. 2) differed in density (being highest for *A*. *severus*, Fig. 4a), proportion of motacú fruits in the diet (being highest for *A*. *severus*, Fig. 4b), and the proportion of dispersed among handled motacú seeds (being highest for *A*. *ararauna*, Fig. 4c). Considering these differences together, the net contribution of macaws to seed dispersal of motacú palms was highest for *A*. *severus*, intermediate for *A*. *ararauna* and lowest for *A*. *glaucogularis* (Fig. 4d).

### Establishment and spatial distribution of immature motacú palms

**Figure 5:**
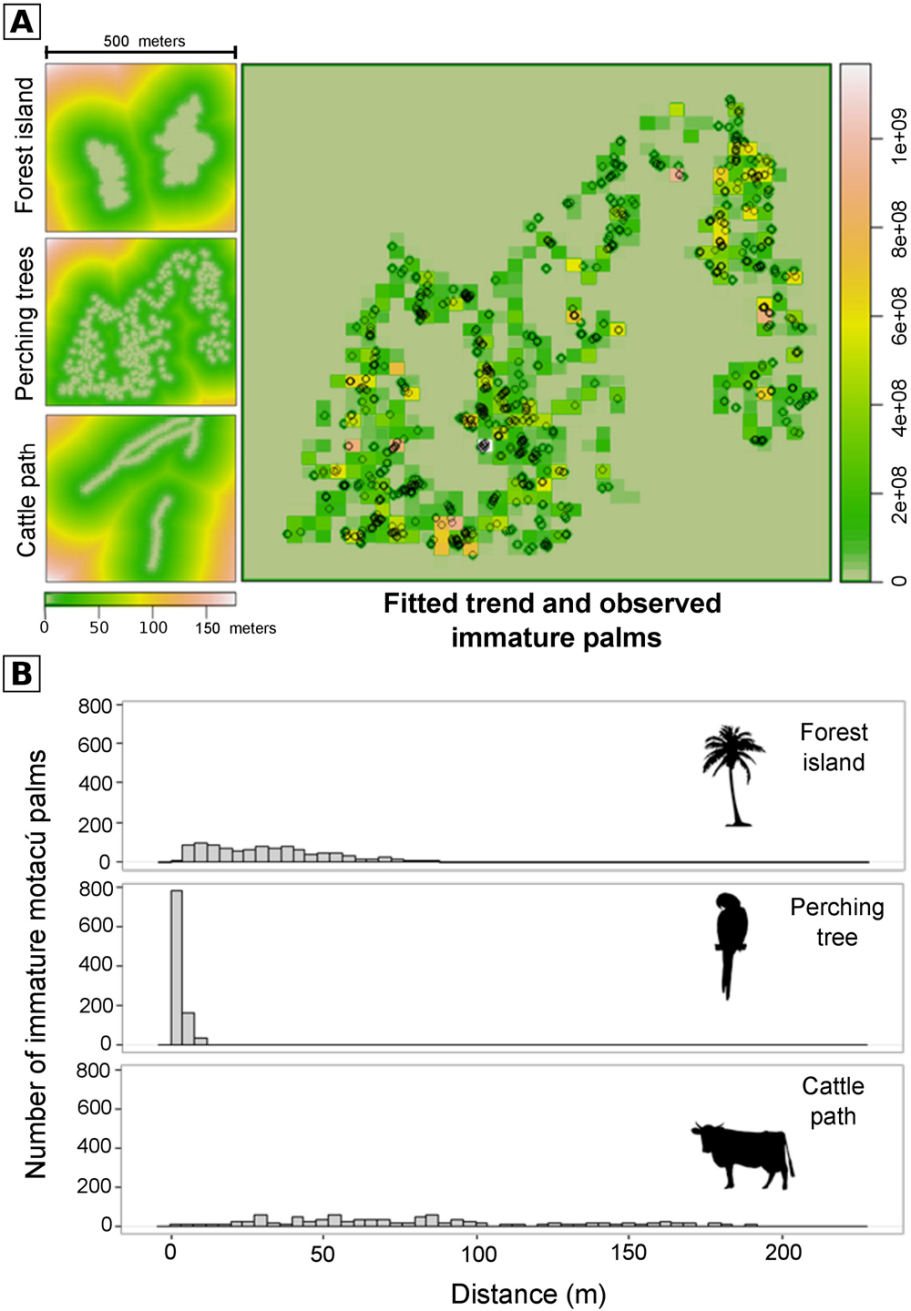
Spatially explicit analysis of motacú palm recruitment. (**A**) On left, spatial covariates (distances to forest islands, perching trees and cattle paths) used to model the spatial distribution of immature motacú palms. The main square is the spatial fitted trend of the best model (including all of the previous covariates), and the observed distribution of immature palms represented with circles. (**B**) Distribution of distances (m) of immature motacú palms to the nearest forest island, perching tree and cattle path.

The model best fitting the observed spatial distribution of the 981 immature motacú palms recorded in the small-scale study area unambiguously indicated the importance of the distances to the nearest perching tree (79% were trees and 21% were dead motacú palms), forest island and cattle path (note ∆AIC > 37 compared to the closest model; Table 2). The probability of finding an immature motacú palm increased as the distances to the nearest perching tree and the nearest forest island decreased (Table 3, Fig. 5). On the contrary, this probability increased with the distance to the nearest cattle path (Table 3, Fig. 5), which may be due to the much higher soil compaction in cattle paths than under perching trees (t = −18.309, df = 97.961, p < 0.0001; mean ± SD soil bulk density (g/cm^3^) was 1.249 ± 0.103 for cattle paths and 0.875 ± 0.101 for perching trees). It is noteworthy that cow faeces were more abundant in resting places called ‘corral’ than in other sites (t = −9.106, df = 27, p < 0.001; mean ± SD faeces/m^2^ was 0.2475 ± 0.138 for corral and 0.0125 ± 0.0136 for other sites). No difference was found in faeces abundance between forest islands, grasslands and cattle paths (all p > 0.05), which may be due to the effect of frequent trampling of faeces in the paths (pers. observ.). The number of seeds excreted by cattle was higher at corral (mean ± SD = 0.045 ± 0.066 seeds/m^2^) than in other sites (0.0014 ± 0.0023 seeds/m^2^) (t = −3.654, df = 27, p = value = 0.001). None of the cattle faeces (n = 1511) was recorded under perching trees.

**Table 2:** Models obtained to relate the spatial distribution of the immature Motacú palms with the spatial covariates related with the dispersers (distance to nearest perching tree for macaws, distance to cattle paths) and the source of seeds (distance to nearest forest island).

Model	AIC	∆ AIC
Distance to nearest perching tree + Distance to nearest forest island + Distance to nearest cattle path	−36361.59	0
Distance to nearest perching tree + Distance to nearest forest island	−36324.23	37.36
Distance to nearest perching tree	−36263.34	98.25
Distance to nearest forest island	−33435.46	2926.13
Null model (Complete Spatial Randomness)	−32870.42	3491.17
Distance to nearest cattle path	−32868.42	3493.17

**Table 3:** Estimates of the variables used in the best spatial model obtained.

Variables (best model)	Estimate	S.E.	Z value	P-value
(Intercept)	21.021	8.32E-02	252.594	<0.0001
Distance to nearest perching tree	−47058.434	1.49E + 03	−31.628	<0.0001
Distance to nearest forest island	−1526.177	1.66E + 02	−9.188	<0.0001
Distance to nearest cattle path	464.178	7.40E + 01	6.275	<0.0001

## Discussion

Our results show that macaws act as legitimate dispersers of the large-seeded motacú palm, always defleshing the fruits and discarding the undamaged seeds at variable distances. Macaws disperse motacú seeds at higher rates than other native frugivorous species and cattle, making a key dispersal contribution due to their relative abundance in the ecosystem and the high proportion of seeds removed. In addition, the spatial distribution of young motacú palms indicates that they are primarily dispersed by macaws. These results evidence that macaws are currently the main primary, short and long distance dispersers of the motacú palm, which is the main biomass-dominant, woody plant in the Beni savanna^44–46, 48^. Macaws act as pervasive seed dispersers, but never as consumers of motacú palm seeds, thus engaging in an ideal plant resource-animal service mutualistic relationship^1^. This contrasts with the previous view of parrots as plant antagonists in their role as mere seed predators, which has been recently revisited and challenged^30–32, 43^. As a consequence of seed dispersal, macaws appear to exert a major influence on the spatial distribution of this foundational species, hence shaping the landscape structure and likely influencing ecosystem function.

The seeds of the motacú palm (but also of other large-seeded tree species) were dispersed by macaws by transporting entire fruits in the beak (stomatochory), as has been recorded for many other plant species dispersed by parrots^30–32, 43, 50^. Macaws drop seeds below mother palms, but most often transport entire fruits to feed on the pulp in distant trees, including dead motacú palms outside forest islands. The observed distances to which seeds were dispersed were variable within and among macaw species, with long-distance dispersal up to 1200 m. Therefore, macaws can be considered important dispersers, making existing islands larger in area through short-distance dispersal and contributing to genetic interchange between distant islands via long-distance dispersal. At a large spatial scale, the average distance between 30 forest islands is 402 m (range: 33–2,485 m, J.A. Díaz Luque unpubl. data), and thus most inter-island distances are within the maximum seed dispersal distances covered by macaws recorded in this study. Macaws thus promote the natural regeneration of the Beni biome and probably increase the connectivity of forest islands, hence improving the resilience of the forest against fragmentation by cattle ranching and other human activities.

With the typical wasteful feeding behavior of parrots, macaws can also exert a very important role as facilitators of seeds for secondary dispersers^30, 51^, although very few secondary dispersal events by jay, squirrel and armadillo species were recorded. In addition, dispersal of seeds through endozoochory^43^ was only recorded for cattle, since they are generally too big (>5 cm) to be swallowed by even the larger frugivorous birds such as curassows or toucans. Monkeys were observed feeding on the pulp and discarding the seed below the fruiting palms, thus promoting secondary dispersal. In addition, large rodent species were recorded feeding on the pulp of dropped motacú fruits, but not dispersing the seeds. Although short-distance primary dispersal by monkeys^52^ and secondary dispersal by rodents^28^ are possible, the relatively short movements of the few recorded secondary dispersers likely result in dispersals at shorter distances than those by macaws^53, 54^. In addition, several of the secondary dispersers of motacú palm can also consume the seeds^55, 56^(pers. observ.).

When dispersal records were weighed by observation effort, the relative contribution to seed dispersal performed by macaws was much higher than that by other species feeding on motacú fruits. This may be a consequence of the higher overall fruit consumption rate of macaws compared with that of other potential primary dispersers, especially squirrels and monkeys, which also showed much lower abundances (authors’ pers. observ.). In addition, the typical mobile and wasting feeding behavior of parrots^30–32^ can contribute to explain the higher motacú seed dispersal rate of macaws compared with other dispersers. There were large differences in the density and the proportion of motacú palm in the diet among macaw species and, consequently, in the proportion of dispersed motacú palm seeds. Macaw density had an especially large influence: the highest net dispersal in quantitative terms^57^ was performed by *A*. *severus*, the species with the highest density. On the other extreme was *A*. *glaucogularis*, despite this species shows similar percentages of motacú in its diet and seed dispersal rates. The extremely low net dispersal contribution of this species is then explained by its rarity in the wild, being a critically endangered species with fragmented and very small populations endemic to the Beni savannas^58^.

Seed dispersal in qualitative terms has been argued to depend on species-specific features of the digestive tract of endozoochorous dispersers^3^, but there is a general lack of information on the factors influencing the quality component of stomatochory. This component can be determined by the distance, microhabitat features and other factors influencing the probability of seedling establishment, which also apply to other dispersal mechanisms^3, 59^. To assess these influences on effective stomatochory requires challenging research including marking individual seeds dispersed by each frugivorous species, and the subsequent monitoring of the fate of seeds. Here, we used a logistically affordable, heuristic approach, to assess disperser impact by considering the distribution of immature motacú palms as a proxy of seedling establishment after presumably being dispersed by stomatochory (macaws) and endozoochory (cattle). The results show that the establishment of immature motacú palms outside forest islands decreased with the distances to the nearest perching tree and forest island border, which suggests actual dispersal events from macaws exclusively using these perching trees outside forest islands. Another large-bodied perching bird, the southern caracara *Caracara plancus*, has been sporadically observed dispersing large seeds of the congeneric *Attalea phalerata* in the Brazilian Pantanal^60^. No southern caracaras were observed performing this behavior during our study despite the fact that this species is common in the area. On the other hand, although cattle showed a quantitatively high dispersal rate of motacú seeds through endozoochory, the presence of immature motacú palms increased with the distance to the nearest main cattle paths. This suggests that seeds excreted by cattle were deposited at high densities close to paths and resting places, where there is high soil compaction, trampling and high herbivory pressure exerted by cattle and therefore it decreases seedling establishment and recruitment^23^.

Most immature motacú palms established outside palm islands were located at distances ranging from 1 to 5 m from the nearest isolated perching tree, thus supporting our predictions that those saplings germinated from seeds that were dropped by macaws after they transported fruits from close islands and consumed the pulp in perching trees. In addition, macaws often drop fruits in flight just after leaving perching sites (n = 30 observations), even when these were previously moved from more distant fruiting plants. This can explain the establishment of immature motacú palms at larger distances from perching trees. The continuous wasting of fruit below or close to fruiting trees during foraging, the transport of entire and partially defleshed fruits to distant trees, and their eventual drooping just after leaving the perching trees or at longer distances in flight has also been recorded in many other parrot species^30–33, 50, 61^. Forthcoming research aimed at evaluating the role of parrots in dispersal of large-seeded plants and its influence on shaping other ecosystem landscapes is needed.

Results of previous and ongoing research indicates that seed dispersal by parrots is a widespread phenomenon involving many plant species^31, 43^. As strong flyers, parrots are particularly efficient at dispersing large seeds of fleshly and dry-fruited plants by transporting them over long distances^30–32^. This kind of dispersal often involves large, heavy and hard seeds that do not require disperser ingestion and defecation or regurgitation to be functionally effective^32^. Motacú and other *Attalea* palms have fruits corresponding to the megafaunal Type I defined by Guimaraes *et al*.^13^ as fleshy fruits 4–10 cm in diameter with up to 5 large seeds (generally >2.0 cm diameter), argued as being adapted for internal dispersal by large extinct terrestrial mammals, thus representing a case of the so-called megafaunal fruit syndrome^12, 13^. The introduction of livestock has been proposed to supply seed dispersal services previously provided by the extinct megafauna given the scarcity, as in our study area, of large extant mammals such as tapirs^40^. However, the role of livestock dispersing motacú palms seems marginal, as assessed from the distribution of immature motacú palms. On the other hand, our study demonstrates that several species of large, highly mobile and still relatively abundant macaws disperse large quantities of seeds to suitable microhabitats where seedling establishment and recruitment as adult palms can be possible and frequent. Thus, although the motacú fruit fully meets the definition of megafaunal syndrome, this species has several extant and reliable primary dispersers represented by large macaws in the Beni biome.

The consideration of parrots, in addition to tapirs, monkeys, carnivore mammals, corvids, squirrels, large rodents and other large vertebrates^14–16, 52, 62, 63^ as legitimate long-distance endozoochorous and especially stomatochorous dispersers of seeds that adjust to the megafaunal syndrome has deep implications in ecology, evolution and conservation of biodiversity. This evaluation is especially important due to the delicate conservation status of many of these species, both dispersers and large-seeded palms and trees^32, 64–68^. In particular, tapirs –considered as the main wild species currently dispersing large seeds in the Neotropics^14, 15, 56, 63^– as well as one third of the parrot species of the world are threatened with extinction^68, 69^. The large-scale population declines and local extinctions of these key dispersers, often due to overexploitation for pet trade and bushmeat^63, 70^, may result in the loss of the ecosystem services provided by them. By acting as primary dispersers and providing access to seeds for secondary dispersers, parrots and other dispersers of large-seeded plants exert a pervasive impact on plant assemblages and ecosystem function^16, 30, 32, 42, 63^. In particular, the close dependence between long-lived large-seeded plants and large seed dispersers suggests that their dispersal and other mutualistic interactions^30^ may lose their current function before the actual disappearance of the species due to human impact^11^. In fact, tapirs are already rare in our studied ecosystem – being absent in our smaller-scale study area (J.A. Díaz, unpubl. data), and the world population of *A*. *glaucogularis* may reach only a few hundreds of individuals^64^. We urge researchers to primarily focus on understanding the role of still extant but rapidly declining large-seed dispersers in the ecology, evolution and conservation of large-seeded plants.

## Methods

### Study system

The study area is located in the savannas of the Beni department in Bolivia (Fig. 1a). This Amazonian ecosystem is characterized by wide areas of seasonal flooded grasslands dotted with forest islands dominated by motacú palm and semi-deciduous groves^44–46^ used historically by indigenous human communities due to the multiple uses they have for this palm^71, 72^, and more recently as pasture for free-range cattle^73^. The *Attalea* genus contains several species of palm trees ranging throughout most of the Neotropical ecosystems, from tropical forests to savannas, generally producing large seeded fruits^74^, and presents taxonomic difficulties due to hybridizations between species^74, 75^. Motacú palms of the Beni savannas were assigned to *A*. *phalerata* ^45, 46, 76^, but recently changed to *A*. *princeps* ^77^. The fruit of motacú palm is an oval-cylindrical drupe rich in lipids, 7–9 cm long and 4–5 cm diameter with yellow flesh when ripe, weighing about 70 g, with a single nut 6–8 cm long and 3–4 cm wide which contains 2–4 seeds^78^ (authors’ unpublished data). The mean annual temperature of the study area is 26 °C, receiving an annual precipitation from 1300 to 2000 mm, with a short dry season from June to September and a wet season the rest of the year^79^. This region holds a high biodiversity with important populations of threatened species^44^, like the critically endangered and endemic Blue-throated macaw *A*. *glaucogularis* ^64^.

### Foraging and seed dispersal observations

Field work was conducted from June to October of 2013. Instead of observing focal motacú palms, we actively searched for large-bodied frugivorous bird (e.g., macaws) and mammal species (e.g., monkeys) across palm patches to increase the probability of finding them, as densities of these species are often small. Once an individual or group was located, we observed them from a distance with telescope and binoculars to record their foraging behavior while avoiding disturbance. Although we focused on the motacú palm, we recorded any other plants consumed by these species to estimate the relative contribution of motacú in their diets. In the case of the three macaw species dispersing motacú (see results), we calculated the proportion of motacú fruits consumed (only pulp) from the total number of fruits (including pulp and seeds of other plant species) consumed. This estimate is very conservative, since the mass consumed from motacú fruits is much larger than that of other fruits due to its much larger size. We also recoded the non-endozoochorous dispersal of other plant species with large fruits and seeds (to estimate the relative dispersal rates of motacú versus other large-seeded plants), excluding potential endozoochory dispersions of small seeds (<5 mm), which requires searching for seeds in faeces^43^. This way, we recorded macaws carrying motacú fruits in their beaks (stomatochory or synzoochory), and the dispersal distances of all observations were measured with the aid of a laser rangefinder^30, 31^. Only a few of these distances (4.3%) were minimum dispersal distances, when fruit-carrying macaws flew out of sight in the vegetation. We also noted if the dispersed fruits were ripe or unripe based on their coloration, as motacú fruits switch from green to brown when ripening, and whether macaws consumed the pulp without damaging the seeds (as only undamaged nuts could contain viable seeds) by later inspecting the consumed fruits dropped and found under the perching trees used by macaws to handle the dispersed fruits (Fig. 3b). We also recorded any other vertebrates, including free-ranging cattle, foraging and eventually dispersing seeds. This way, we recorded daylight (during 6–10 h AM and 16–19 h PM) foraging and dispersal behaviors during 420 hours of field work. These observations were made in 25 different locations along survey transects covering a wide area of 26,383 km² (Fig. 1a).

To attempt recording secondary dispersal events by more elusive, scarce or nocturnal potential dispersers like ground dwelling mammals, including rodents, carnivore mammals, etc^14, 28, 80^, we used camera traps (2,320 cumulative hours, camera model: Bushnell 6 MP Trophy Cam Essential). These cameras were placed at ground level under fleshy-fruited trees with presence of fallen mature motacú fruits to increase the chance of detection of secondary dispersers in 30 different sites (overlapping with the 25 observation sites). We also surveyed these sites with flashlights at night (60 hours) to take into account other arboreal-dwelling potential dispersers such as nocturnal monkeys. To control for differences in sampling effort between the three methodologies used, we obtained dispersal rates of motacú seeds for each frugivorous species by dividing the number or dispersal events recorded by the number of hours invested on the methodology from which the species was recorded (i.e., 420 h for diurnal observations, 60 h for nocturnal observations, and 2,320 h for full-day camera trapping).

### Macaw density estimation

To assess the net dispersal contribution^57^ of the three macaw species (*A*. *ararauna*, *A*. *glaucogularis* and *A*. *severus*) that acted as motacú seed dispersers, we estimated their densities using count data from road-side survey transects^30^ conducted across the Beni savannas biome (Fig. 1a). Surveys were conducted by two observers driving a car at slow speed (20–40 km/h) through a total of 734 km of unpaved roads, stopping each time parrots were heard or sighted to identify the species, flock size, and distance to observer using a laser rangefinder^30^. Detailed methods of abundance estimation are provided in Supporting Information.

### Spatial data collection and analysis

We assessed factors associated to the spatial distribution of immature motacú palms as a proxy for palm recruitment^81, 82^. This was done over a square area of 12.2 ha, including two isolated forest islands (of 1.57 and 0.80 ha) dominated by this palm, surrounded by open seasonal flooded grassland (Fig. 1b). Through an exhaustive search, we recorded the geographical coordinates of all the motacú palms that were outside the boundaries of the forest islands within the 12.2 ha survey area, and categorized them into two age classes according to their height: adults and immature palms (<2 m, including seedlings and recently recruited palms). Apart from the two target forest islands, the closest forest island in the area was 850 m away from the nearest studied forest island, so most of the motacú palms were presumably dispersed from one of these two forest islands (see Fig. 2c showing that most dispersal distances were <850 m).

To evaluate palm recruitment, we focused on macaws and cattle, the main dispersers in terms of number of motacú seeds moved during foraging (see results). Macaws often use perching trees to rest during the day or to manipulate food items gathered in other trees^30–32^. Specifically, macaws usually transport motacú palm fruits in the beak and fly to a perching tree where they manipulate and deflesh the fruit, dropping the seed without damage^49^ (see results). We located all trees grown in the grassland areas (Fig. 1b, through an exhaustive search) and isolated from the forest islands that were used as perches by macaws (the few mature motacú palms used as perching trees were not included in the analysis), but not by other non-avian potential dispersers such as monkeys since they are reluctant to leave the forest islands. Through an exhaustive search, we located all the main paths used frequently by cattle during daily movements (Fig. 1b), where excreted defleshed seeds were abundant. In addition, to better understand how livestock used the space in the study area (Fig. 1b), we recorded all cattle faeces (noting if there were seeds inside) along transects 100 m long and 2.5 m wide on each side in the different habitats: forest islands, grasslands, cattle paths (distinguishing between along grasslands or along forest islands), under perching trees, and in concentration places (called corral in the study area) where cattle pass long periods resting. We surveyed 8 transects in forest islands, 8 in grasslands, 9 in cattle paths and 4 in corrals within the study area.

To determine the importance of dispersers in seed survival and recruitment, we modeled the spatial distribution of all immature motacú palms as a result of seedling establishment out of the two forest islands. Thus, the spatial distribution of dispersed seeds becoming immature palms was analyzed fitting Spatial Point Pattern Models using the *spatstat* package^83^ for R program^84^, considering the UTM coordinates of each sapling as the dependent variable. We considered the effect of spatial covariates related with the dispersers’ activity, such as the distance (in meters) to perching trees potentially used by macaws and distance to cattle paths. We also included the straight-line distance (in meters) to the nearest forest island as the presumable source of the seeds producing these immature palms. Elevation could be another important covariate, but there were no appreciable elevation differences in the area, except on finer scale^85^ where some big trees and termite mounds have elevated (less than 0.5 m) the soil, and therefore it was not included in the models. The models were compared using AIC (Akaike Information Criterion), with lower values indicating a better fit to data^86^, and then contrasted against a null model based on Complete Spatial Randomness, generated by a uniform Poisson point process.

Soil compaction in the cattle paths (50 sites) and under perching trees (50 sites) was measured to assess whether this microhabitat component of locations where seeds are frequently deposited could have an influence on seed survival and recruitment as immature palms. We used the core sampling method^87^ to measure soil bulk density (i.e., the dry soil mass in a given soil volume). We assumed that more compacted soils represent low-quality dispersal sites precluding germination and sapling establishment^23^.

## Electronic supplementary material

Supplementary Information
