# Supplementary material for: Seed dispersal by macaws shapes the landscape of an Amazonian ecosystem
Source: Sci Rep. 2017 Aug 7;7:7373. doi: 10.1038/s41598-017-07697-5 (PMC5547140; doi:10.1038/s41598-017-07697-5)
Supplement: Supplementary file 1 — Supplementary Information [file 41598_2017_7697_MOESM1_ESM.pdf]

Seed dispersal by macaws shapes the landscape of an Amazonian ecosystem

Adrián Baños-Villalba[1], Guillermo Blanco[2], José A. Díaz-Luque[3], Francisco V. Dénes[4], Fernando Hiraldo[4] and José L. Tella[4]

Macaw density estimation. The numbers of birds observed is the product of their underlying abundance and detectability [1], assumed to be primarily a function of distance-to-observer, and thus we employed a hierarchical distance sampling technique [2] adapted for line transect surveys with "sighting" (i.e. non-perpendicular) distance data (Dénes et al, *in prep)* to model detection probability for each species. Essentially, the method consists of using point-transect geometry to estimate the detection function − thus accounting for non-perpendicular distance measurements. Simulations show that estimates of detection function parameters and derived abundance obtained from "sighting" distance data with this method have negligible bias compared to those generated with line-transect distance sampling geometry (Dénes et al, *in prep*). Detection models were fitted using the *unmarked* package [3] for program R [4]. Following recommendations in Buckland et al. (2001), we defined maximum detection distance limits (right truncation) for each species (w_{sp}) by excluding the 5% farthest detections and continued the analysis with the data from the remaining 95% records. Distances were recorded on a continuous scale, but after exploratory analysis we decided to group them into distance classes to facilitate fitting of the detection function. Distance intervals were defined as one tenth of w_{sp}, to reduce loss of precision [5]. We compared detection models with different key functions (half-normal and negative exponential), and selected the best model for each species based on the Akaike Information Criterion (AIC). We derived the specific average detection probabilities (p) by integrating the detection functions over w_{sp}.

Detectability-corrected estimates of abundance for each species were obtained by dividing the total counts by their specific p. For *A. glaucogularis*, however, the very low number of contacts with distance measurements (n=3) makes it impossible to fit a detection function for this species. Therefore, to infer density of this species, which is close to extinction in the wild [6], we assumed that its detection probability was similar to that of *A. ararauna*, which has similar size, coloration and habits [7]. Densities were calculated by dividing the detectability-corrected estimates of abundance by the area surveyed [individuals/($km_{transects} \cdot 2w_{sp}$)]. The number of detections, distance intervals, the 95% maximum detection distances (w_{sp}) used for each species, and the estimated detection functions, probabilities and densities, with 95% confidence intervals are shown in Table S1 and Fig. S1.

References

1. Dénes, F. V., Silveira, L. F. & Beissinger, S. R. Estimating abundance of unmarked animal populations: accounting for imperfect detection and other sources of zero inflation. *Methods Ecol. Evol.* **6,** n/a-n/a (2015).

2. Royle, J. A., Dawson, D. K. & Bates, S. Modeling abundance effects in distance sampling. *Ecology* **85,** 1591–1597 (2004).

3. Fiske, I. & Chandler, R. unmarked : An R Package for Fitting Hierarchical Models of Wildlife Occurrence and Abundance. *J. Stat. Softw.* **43,** 1–23 (2011).

4. R Core Team. R: A Language and Environment for Statistical Computing. (2015). at <http://www.r-project.org/>

5. Kéry, M. & Royle, J. A. in *Applied Hierarchical Modeling in Ecology* 393–461 (Academic Press., 2016).

6. Hesse, A. J. & Duffield, G. E. The status and conservation of the Blue-Throated Macaw Ara glaucogularis. *Bird Conserv. Int.* **10,** 255–275 (2000).

7. Parr, M. & Juniper, T. *Parrots: A Guide to Parrots of the World*. (Bloomsbury Publishing, 2010).

Table S1. Counts, distance intervals, the 95% maximum detection distances (w_{sp}) used for each species, and the estimated detection probabilities and densities, with 95% confidence intervals. Negative-exponential models of the detection function were selected over half-normal model for both *A. ararauna* (ΔAIC = 20.94) and *A. severus* (ΔAIC = 61.75).

Species	Count	Distance interval (m)	*w* (m)	*p*	Density (ind./ha)	$CL_{0.05}$	$CL_{0.95}$
Ara ararauna	151	28.35	283.5	0.179	0.011	0.010	0.013
Ara severus	411	22.14	221.4	0.179	0.040	0.043	0.037
Ara glaucogularis	9	-	283.5	0.179*	0.00068	0.00076	0.00060

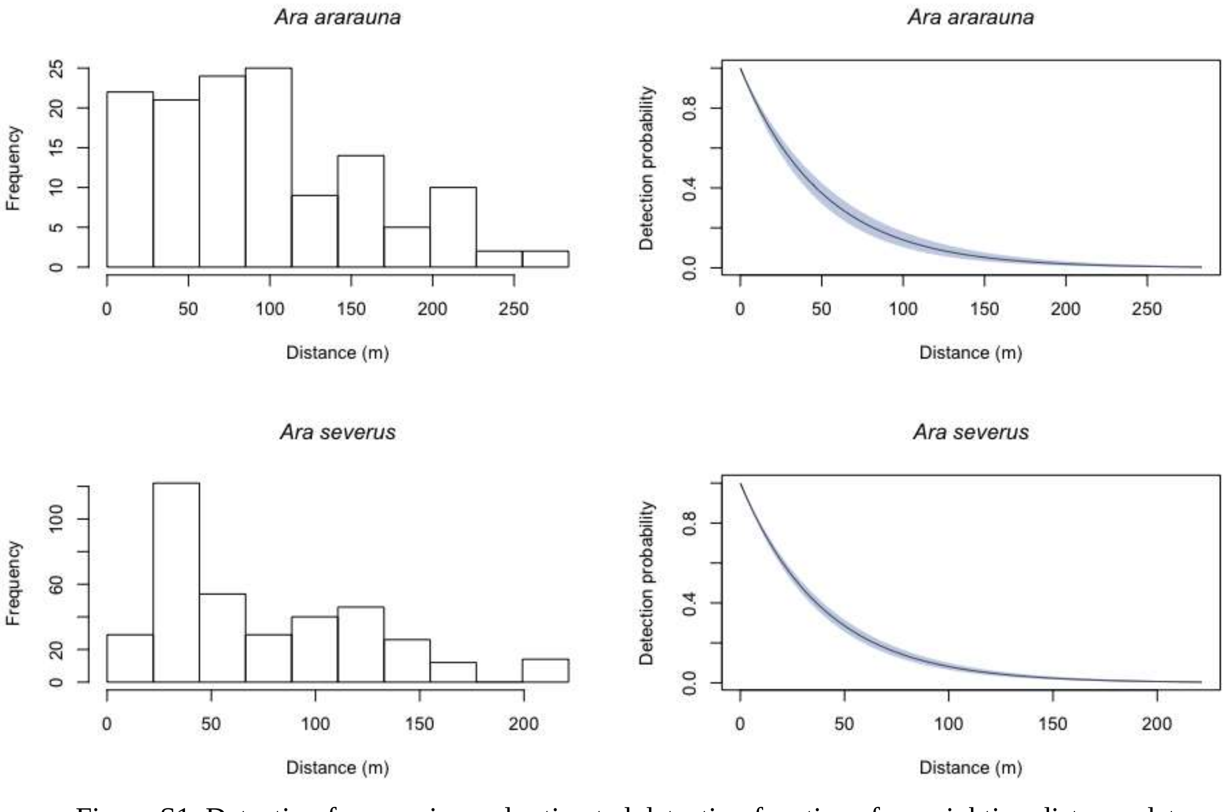

Figure S1. Detection frequencies and estimated detection functions from sighting distance data for *Ara ararauna* and *A. severus,* with 95% confidence interval.
